# A Proof of Concept for the Use of Lung Ultrasound in the Diagnostic Approach of Pulmonary Hypertension

**DOI:** 10.1016/j.chpulm.2025.100225

**Published:** 2025-10-23

**Authors:** Benoit Aguado, Thomas Lacoste-Palasset, Florent Favard, Sylvain Palat, Victor Aboyans, Marc Humbert, David Montani

**Affiliations:** a Department of Respiratory Medicine, University of Limoges, Limoges University Hospital, Limoges, France; b INSERM UMR_S 999 “Pulmonary hypertension: pathophysiology and novel therapies (HPPIT), Marie Lannelongue Hospital, Le Plessis-Robinson, France; c Department of Respiratory and Intensive Care Medicine (FHU André Cournand, ERN-LUNG), Université Paris–Saclay, AP-HP, Bicêtre Hospital, Le Kremlin Bicêtre, France; d Department of Internal Medicine A, Dupuytren 2 University Hospital, Limoges, France; e Department of Cardiology, Limoges University, Dupuytren University Hospital, and EpiMaCT, Inserm1094/IRD270, Limoges, France

To the Editor:

Pulmonary hypertension (PH) is a hemodynamic condition defined by right heart catheterization (RHC) as a mean pulmonary arterial pressure > 20 mm Hg. PH is categorized based on pulmonary vascular resistance (PVR) and pulmonary arterial wedge pressure (PAWP): precapillary PH is defined as PVR > 2 Wood units (WU) and PAWP ≤ 15 mm Hg, isolated postcapillary PH is defined as PVR ≤ 2 WU and PAWP > 15 mm Hg, and combined precapillary and postcapillary PH is defined as PVR > 2 WU and PAWP > 15 mm Hg.^1^ Precise hemodynamic classification is critical because misclassification may lead to inappropriate treatment. For example, the use of pulmonary arterial hypertension (PAH)-approved drugs in postcapillary PH has been associated with worse outcomes and increased mortality.^2^ PAWP, measured by RHC, is the most common way to estimate left ventricular end-diastolic pressure and is pivotal in distinguishing precapillary and postcapillary PH.^3^

Lichtenstein et al^4^ has shown that an A-line predominance on lung ultrasound (LUS) correlated with low PAWP, whereas an abrupt transition to a B-line predominance indicated a wet lung, with increased extravascular lung water and elevated PAWP. These LUS profiles were initially validated in critically ill ICU patients in acute settings,^4^ and are now routinely integrated in the cardiology field to assess pulmonary congestion in heart failure and stress echocardiography.^5^^,^^6^ However, the specific role of LUS in the diagnosis and characterization of PH remains poorly investigated. Here, we present a retrospective study investigating whether an A-line predominance can reliably predict normal PAWP, and thereby support the diagnosis of precapillary PH.

## Methods

We retrospectively analyzed data from adult patients enrolled in the Pulmotension registry (Commission Nationale de l’Informatique et des Libertés No. 842063) from the Limoges Competence Center between May 1, 2020, and May 1, 2021. Inclusion criteria were the following: (1) confirmed PH diagnosed by RHC, and (2) LUS and echocardiography performed before RHC.

Demographic, clinical, biological, functional, hemodynamic, and echocardiographic data were collected. LUS was performed as part of standard care before RHC, using a Philips CX50 system with an S5-1 probe, and was interpreted blinded to the patient’s medical history and RHC results. Anterior bedside LUS in emergency points were examined, with each lung divided into 4 points of interest.^4^ Examinations were acquired in B-mode, with transmit frequency of 5 MHz, harmonics off, and the focal zone placed at the pleural line. Patients were categorized depending on LUS pattern: A-line predominance was defined as the presence of A, A/B, or A′ profiles without B-lines. Conversely, B-line predominance included B or B′ profiles characterized by B-lines. B profile corresponds to the presence of ≥ 3 B-lines per intercostal space in multiple bilateral lung zones, whereas a B′ profile refers to a B profile without visible lung sliding.

The primary outcome was to assess the diagnostic performance of A-line predominance in predicting a PAWP ≤ 15 mm Hg. Echocardiographic left atrial dilation, defined as a left atrial volume index > 34 mL/m^2^, was used as a comparator. Statistical analyses were performed using the software R version 3.6.1 (R Foundation for Statistical Computing). Diagnostic accuracy was evaluated using sensitivity, specificity, positive and negative predictive values, and positive likelihood ratio (LR^+^) and negative likelihood ratio (LR^-^) with their 95% CIs. Results are expressed as median (interquartile range) for continuous variables and frequency and percentage for categorical variables. Between-group comparison was performed with nonparametric Mann-Whitney *U* test and categorical variables with Fisher exact test. A bilateral *P* < .05 was considered significant.

## Results

A total of 30 patients were included: 19 exhibited A-line predominance and 11 exhibited B-line predominance. Clinical and hemodynamic characteristics are detailed in Table 1.

**Table 1:** Characteristics of Patients

Characteristic	All Patients (N = 30)	A-Line Predominance (n = 19)	B-Line Predominance (n = 11)	No. of Patients	*P* Value
Demographics					
Age, y	65.0 (60.5-75.0)	65.0 (59.0-75.0)	65.0 (63.5-78.0)	30	.42
Female	18 (60)	11 (58)	7 (64)	30	.99
BMI, kg/m^2^	29.0 (24.0-30.5)	27.0 (24.0-30.0)	32.0 (31.2-36.5)	23	**< .01**
Tobacco use	10 (37)	6 (32)	4 (50)	27	.41
Atrial fibrillation	8 (27)	1 (5.3)	7 (64)	30	**< .01**
Chronic renal failure^a^	6 (20)	0 (0)	6 (55)	30	**< .001**
COPD	8 (27)	6 (32)	2 (18)	30	.67
Interstitial lung disease	5 (17)	2 (11)	3 (27)	30	.33
Coronary artery disease	9 (30)	2 (11)	7 (64)	30	**< .01**
Diabetes	2 (7)	2 (11)	0 (0)	30	.52
Systemic hypertension	18 (60)	8 (42)	10 (91)	30	**.018**
Functional and biological parameters					
PH group 1, 2, 3, 4	10/10/7/3	9/1/6/3	1/9/1/0	30	**< .001**
NHYA I-II vs III-IV	12 (40)	8 (42)	4 (36)	30	.99
NT-pro-BNP < 300 ng/L	14 (56)	10 (53)	4 (67)	25	.66
Echocardiography					
E/A	0.75 (0.70-0.97)	0.75 (0.71-1.0)	0.70 (0.70-0.93)	20	.73
Left atrial dilatation^a^	13 (45)	5 (28)	8 (73)	23	**.027**
Pulmonary function tests					
FVC %	78.5 (61.5-95.8)	78.5 (60.5-90.5)	86.0 (74.0-98.9)	24	.61
FEV_1_ %	79.5 (48.5-90.2)	79.5 (47.5-90.0)	72.5 (55.2-92.0)	24	.92
FEV_1_/FVC	0.730 (0.650-0.780)	0.730 (0.650-0.780)	0.715 (0.670-0.745)	24	.82
Dlco %	43.0 (25.0-56.0)	47.0 (26.0-54.0)	34.0 (26.0-52.5)	19	.79
Hemodynamic parameters					
RAP, mm Hg	7.50 (6.00-10.0)	6.0 (5.00-8.00)	10.0 (8.00-11.0)	30	**< .001**
mPAP, mm Hg	32.5 (29.2-38.8)	31.0 (24.5-38.5)	36.0 (30.0-39.5)	30	.39
CO, L/min	4.6 (4.0-6.0)	4.6 (4.2-5.9)	4.8 (4.1-6.4)	30	.48
CI, L/min/m^2^	2.70 (2.40-3.30)	2.50 (2.35-3.26)	2.90 (2.65-3.60)	25	.54
PAWP, mm Hg	13.5 (10.2-18.0)	12.0 (9.25-14.0)	19.0 (17.5-19.5)	35	**< .001**
PAWP ≤ 15 mm Hg	19 (63)	18 (95)	1 (9)	30	**< .001**
PVR, WU	3.65 (2.55-5.37)	3.90 (2.55-6.55)	3.20 (2.60-4.35)	30	.37
Hemodynamic classification				30	**< .001**
Postcapillary^b^	11 (37)	1 (5)	10 (91)		
Precapillary^c^	19 (63)	18 (95)	1 (9)		
Fluid challenge test performed	4 (13)	1 (5.3)	3 (27)	30	**.013**
Positive fluid challenge test	3 (75)	0 (0)	3 (100)	4	.25

Data are presented as median (interquartile range), No. (%), No., or as otherwise indicated. Bold font in *P* value indicates significant results of the comparison between A-line and B-line predominances. CI = cardiac index; CO = cardiac output; Dlco = diffusing capacity of the lung for carbon monoxide; E/A = ratio of early (E) to late (A) mitral inflow velocities; mPAP = mean pulmonary arterial pressure; NT-proBNP = N-terminal pro-B-type natriuretic peptide; NYHA = New York Heart Association; PAWP = pulmonary artery wedge pressure; PH = pulmonary hypertension; PVR = pulmonary vascular resistance; RAP = right atrial pressure; WU = Wood units.

a Defined by left atrial volume index > 34 mL/m^2^.

b Nine patients had mixed postcapillary and precapillary pulmonary hypertension.

c Among patients with precapillary PH, 10 were classified with group 1 pulmonary arterial hypertension, 7 had group 3 PH, and 3 had group 4 PH.

Among patients with B-line predominance, 95% had a PAWP > 15 mm Hg, compared with only 5.3% in the A-line predominance group (*P* < .001). No significant differences were observed in mean pulmonary arterial pressure, cardiac output, cardiac index, or PVR between the 2 groups. Postcapillary PH was significantly more frequent in the B-line predominance group (95% vs 9.1%), whereas precapillary PH was predominant in the A-line group (91% vs 5.3%; *P* < .001). Fluid challenge was more often performed and revealed more frequently left ventricular diastolic dysfunction in the B-line predominance group, respectively (27% vs 5.3%; *P* = .013 and 27% vs 0%; *P* = .041).

LUS A-line predominance demonstrated high diagnostic accuracy for identifying a PAWP ≤ 15 mm Hg, with a sensitivity of 94.7% (95% CI, 74.0-99.9) and a specificity of 90.9% (95% CI, 58.7-99.8). The positive predictive value and negative predictive value were 94.7% and 90.9%, respectively. The LR^+^ and LR^-^ of an A-line predominance were 10.4 (95% CI, 1.6-67.7) and 0.06 (95% CI, 0.01-0.39), respectively. In contrast, the diagnostic accuracy of left atrial dilatation was more limited, with an LR^+^ of 2.7 (95% CI, 1.0-7.3) and an LR^-^ of 0.38 (95% CI, 0.17-0.87) (Table 2).

**Table 2:** Diagnostic Performance of A-Line Predominance for a Pulmonary Artery Wedge Pressure ≤ 15 mm Hg

Variables	Sensibility, % (95% CI)	Specificity, % (95% CI)	Positive Predictive Value, % (95% CI)	Negative Predictive Value, % (95% CI)	Positive Likelihood Ratio, No. (95% CI)	Negative Likelihood Ratio, No. (95% CI)	Disease Prevalence,^a^ % (95% CI)
A-line predominance, n = 30	94.7 (74.0-99.9)	90.9 (58.7-99.8)	94.7 (73.5-99.2)	90.9 (59.5-98.6)	10.4 (1.6-67.7)	0.06 (0.01-0.39)	63.3 (43.9-80.1)
LAVI ≤ 34 mL/m^2^, n = 29	72.2 (46.5-90.3)	72.7 (39.0-94.0)	81.2 (61.3-92.2)	61.5 (41.1-78.6)	2.7 (1.0-7.3)	0.38 (0.17-0,87)	62.1 (42.3-78.6)

LAVI = left atrial volume index.

a Precapillary pulmonary hypertension.

## Discussion

To our knowledge, this is the first study to evaluate the diagnostic performance of LUS and A-line predominance in differentiating precapillary from postcapillary PH. Our findings suggest that the presence of an A-line predominance is predictive of a PAWP ≤ 15 mm Hg and may serve as a noninvasive surrogate for identifying precapillary PH.

Although RHC remains the criterion standard for hemodynamic classification, a diagnostic gray zone exists when PAWP values fall between 12 and 18 mm Hg.^3^^,^^7^ The accuracy of PAWP measurement may be compromised in conditions involving intrathoracic pressures such as chronic obstructive disease or obesity.^8^ In such settings, end-expiratory PAWP values may either underestimate or overestimate true left atrial pressure, contributing to diagnostic uncertainty, particularly when PAWP values lie close to the 15 mm Hg threshold. Alternative approaches, including the use of esophageal pressure monitoring to estimate pleural pressure, have been proposed to enhance PAWP accuracy, but are invasive and not widely available.^9^

LUS offers a rapid, bedside, and noninvasive method to assess pulmonary congestion by detecting interstitial syndrome through the presence of B-lines. An A-line predominant pattern suggests absence of interstitial fluid and correlates with normal left heart filling pressures. In our study, A-line predominance showed high diagnostic accuracy, with sensitivity and specificity > 90%, and an LR^+^ of 10.4, supporting its utility in ruling in precapillary PH. In contrast, traditional echocardiographic markers such as left atrial volume index demonstrated lower discriminative power (LR^+^ = 2.7), likely due to overlap between etiologies and delayed anatomic changes. Integrating LUS at the time of RHC could thus enhance diagnostic confidence and potentially reduce the need for provocative testing, such as fluid challenge, in selected patients.

Previous studies have explored combined diagnostic strategies. D’Alto et al^5^ proposed a composite approach using echocardiography, LUS, and dynamic variation in B-lines after fluid challenge to differentiate PAH from PH due to heart failure with preserved ejection fraction. In this study, the variation in number of B-lines after fluid challenge improved the diagnostic accuracy of an echocardiographic score to distinguish PAH from PH due to heart failure with preserved ejection fraction.^5^ Although promising, this method required a cumulative scoring system based on necessity of fluid challenge to identify the variation in number of B-lines across multiple zones adds complexity, which may limit its routine clinical application. In contrast, our approach focuses on a static, easily interpretable LUS pattern performed immediately before or during RHC, increasing its feasibility in high-volume or resource-limited settings.

Nonetheless, several limitations must be acknowledged. First, this is a retrospective single-center study, which should be validated in a prospective multicenter setting. Second, the small sample size limits the statistical power and precision of the diagnostic estimates, as reflected by the wide CIs. Third, all LUS examinations were performed by a single experienced operator, precluding assessment of interobserver reproducibility. Finally, false-positive B-line patterns may arise in patients with interstitial lung disease or pulmonary fibrosis, conditions that mimic pulmonary edema without necessarily reflecting elevated left-sided heart filling.^10^

Despite these limitations, this study highlights the potential value of LUS as a complementary tool to RHC for the classification of PH. Incorporating a simple A-line/B-line assessment into the diagnostic algorithm may help guide the bedside diagnostic approach, particularly in ambiguous cases or when invasive measures yield equivocal results. Future prospective studies in larger and more diverse cohorts, including reproducibility assessments, are warranted to validate these findings and to define the role of LUS in PH workup more precisely.

## Financial/Nonfinancial Disclosures

The authors have reported to *CHEST Pulmonary* the following: B. A. reports honoraria for lectures and educational events from GSK, Chiesi, Sanofi, and Boehringer Ingelheim. M. H. reports consulting fees from 35 Pharma, AOP Orphan, Chiesi, Ferrer, Gossamer, Inhibikase, Johnson & Johnson, Keros, Liquidia, Merck, Novartis, Regeneron, Respira, Pfizer, Pulmovant, and United Therapeutics; lecture fees from Merck; participation in advisory boards for 35 Pharma, Aerovate, Janssen, Inhibikase, Keros, Merck, Novartis, and United Therapeutics; and institutional research support from Gossamer and Merck. D. M. reports consulting fees from Ferrer and Merck MSD; honoraria for lectures and educational activities from Bayer, Boehringer, Chiesi, GSK, Jazz Pharmaceuticals, Ferrer, and Merck MSD; and institutional research support from Gossamer and Merck. T. L.-P. reports funding for travel and housing to assist the 2023 ERS Congress. None declared (F. F., S. P., V. A.).

## References

[1] Humbert M., Kovacs G., Hoeper M.M. (2022). 2022 ESC/ERS guidelines for the diagnosis and treatment of pulmonary hypertension. Eur Heart J.

[2] Hoeper M.M., Oerke B., Wissmüller M. (2024). Tadalafil for treatment of combined postcapillary and precapillary pulmonary hypertension in patients with heart failure and preserved ejection fraction: a randomized controlled phase 3 study. Circulation.

[3] Kovacs G., Bartolome S., Denton C.P. (2024). Definition, classification and diagnosis of pulmonary hypertension. Eur Respir J.

[4] Lichtenstein D.A., Mezière G.A., Lagoueyte J.-F., Biderman P., Goldstein I., Gepner A. (2009). A-lines and B-lines: lung ultrasound as a bedside tool for predicting pulmonary artery occlusion pressure in the critically ill. Chest.

[5] D’Alto M., Liccardo B., Di Maio M. (2023). Lung ultrasound, echocardiography, and fluid challenge for the differential diagnosis of pulmonary hypertension. J Am Soc Echocardiogr.

[6] Platz E., Systrom D., Leopold J.A. (2024). Pulmonary congestion in patients with pulmonary arterial hypertension? New insights from lung ultrasound. Eur J Heart Fail.

[7] Maron B.A., Bortman G., Marco T.D. (2024). Pulmonary hypertension associated with left heart disease. Eur Respir J.

[8] LeVarge B.L., Pomerantsev E., Channick R.N. (2014). Reliance on end-expiratory wedge pressure leads to misclassification of pulmonary hypertension. Eur Respir J.

[9] Khirfan G., Melillo C.A., Al Abdi S. (2022). Impact of esophageal pressure measurement on pulmonary hypertension diagnosis in patients with obesity. Chest.

[10] Lichtenstein D.A. (2019). Current misconceptions in lung ultrasound: a short guide for experts. Chest.

